# Smoking Cessation among Low-Socioeconomic Status and Disadvantaged Population Groups: A Systematic Review of Research Output

**DOI:** 10.3390/ijerph120606403

**Published:** 2015-06-08

**Authors:** Ryan J. Courtney, Sundresan Naicker, Anthony Shakeshaft, Philip Clare, Kristy A. Martire, Richard P. Mattick

**Affiliations:** 1 National Drug and Alcohol Research Centre (NDARC), University of New South Wales, Sydney, NSW 2052, Australia; E-Mails: s.naicker@unsw.edu.au (S.N.); a.shakeshaft@unsw.edu.au (A.S.); p.clare@unsw.edu.au (P.C.); k.martire@unsw.edu.au (K.A.M.); r.mattick@unsw.edu.au (R.P.M.); 2 School of Psychology, University of New South Wales, Sydney, NSW 2052, Australia

**Keywords:** smoking cessation, social class, socio-economic factors, poverty, review, intervention, homeless persons, indigenous population, mental disorders, prisoners

## Abstract

*Background*: Smoking cessation research output should move beyond descriptive research of the health problem to testing interventions that can provide causal data and effective evidence-based solutions. This review examined the number and type of published smoking cessation studies conducted in low-socioeconomic status (low-SES) and disadvantaged population groups. *Methods*: A systematic database search was conducted for two time periods: 2000–2004 (TP1) and 2008–2012 (TP2). Publications that examined smoking cessation in a low-SES or disadvantaged population were coded by: population of interest; study type (reviews, non-data based publications, data-based publications (descriptive, measurement and intervention research)); and country. Intervention studies were coded in accordance with the Cochrane Effective Practice and Organisation of Care data collection checklist and use of biochemical verification of self-reported abstinence was assessed. *Results*: 278 citations were included. Research output (*i.e*., all study types) had increased from TP1 27% to TP2 73% (*χ*² = 73.13, *p* < 0.001), however, the proportion of data-based research had not significantly increased from TP1 and TP2: descriptive (TP1 = 23% *vs.* TP2 = 33%) or intervention (TP1 = 77% *vs.* TP2 = 67%). The proportion of intervention studies adopting biochemical verification of self-reported abstinence had significantly decreased from TP1 to TP2 with an increased reliance on self-reported abstinence (TP1 = 12% *vs.* TP2 = 36%). *Conclusions*: The current research output is not ideal or optimal to decrease smoking rates. Research institutions, scholars and funding organisations should take heed to review findings when developing future research and policy.

## 1. Introduction

Smoking rates have declined in most developed countries [1,2], but inequalities in smoking rates have persisted, or increased, over time [3,4,5,6]. Data collected from the Global Tobacco Surveillance System [7], the World Health Organisation (WHO) STEPwise approach to surveillance (STEPS) program [8] and Health Surveys [9] have demonstrated that the frequency, quantity and modes of tobacco smoking are not equally distributed within or across countries or population groups. The cumulative prevalence of tobacco consumption is often highest among those with low educational attainment and lower income levels [10]. The smoking rate among individuals with a mental illness is nearly twice that of adults without mental illness [11]. Similarly for other disadvantaged population groups smoking rates are markedly higher compared to the general population, e.g., homeless persons 70 percent [12,13], Indigenous persons 50 percent [14,15], and prisoners between 50 to 83 percent [16].

Reducing smoking rates among low-socioeconomic status (low-SES) and disadvantaged groups is critical to improving life expectancy and reducing health inequalities [17]. Using indirect estimation techniques to attribute mortality caused by smoking across four countries (Australia, UK, Canada, and the US), smoking accounts for nearly half of excess mortality among men aged 35–69 years within the lowest SES stratum [18]. In the UK, over half of the difference in survival to age 70 years between the highest and lowest social classes is attributable to the higher smoking prevalence in the lowest socioeconomic quintile [17,19]. The economic costs of smoking are high among low-SES and disadvantaged groups. Most recent estimates using the WHO’s Economics of Tobacco Toolkit indicate that the economic cost of smoking for people with mental illness in the UK was £2.34 billion from 2009 to 2010 [20]. This high cost and burden of disease attributable to tobacco smoking has led to calls for the “*development and implementation of smoking cessation interventions*” to be a “*high economic and clinical priority*” among low-SES and disadvantaged population groups, particularly smokers with mental disorders [20].

Tobacco consumption is a well-elucidated modifiable risk factor and its cessation may significantly reduce both the absolute and relative risk of disease burden and mortality across all population groups.

Tobacco surveillance activities have provided useful information on the implementation of tobacco control efforts and World Health Surveys have identified complex patterns of tobacco use and social determinants of smoking [10]. However, to improve our global knowledge base, it is important that appropriate interventions are targeted, implemented and evaluated. The WHO Framework Convention on Tobacco Control (WHO FCTC) was ratified in 2005 by 168 WHO members states and has identified as a key priority the need for signatory countries to: “*design and implement effective programmes aimed at promoting the cessation of tobacco use*” [21,22]. All countries included in this review (at the exception of two countries *i.e.*, China and Taiwan) were signatories to the WHO FCTC, which aims to implement an internationally coordinated strategy to both reduce the uptake and increase cessation. Disparities in smoking prevalence highlight the need for strategies to address both smoking initiation and increasing smoking cessation among low-SES and disadvantaged groups [23]. The focus of this review is to reach a critical endpoint of identifying research output related to smoking cessation among low-SES and disadvantaged groups.

Two consistently used measures to quantify the contribution of research applied to other health-related fields are: the number of publications, and the type of research design (e.g., measurement, descriptive, intervention and evaluation of intervention effects) [24,25]. For the purpose of this review, these same measures were used in order to systematically quantify and evaluate smoking cessation research output regarding low-SES and disadvantaged groups.

This review will examine research output across countries to identify any differences in patterns of research output across countries, or in particular whether some countries are more or less likely to focus on intervention research that has the potential to reduce smoking rates among low-SES or disadvantaged groups. This is a difficult question to answer, as there are numerous dimensions that could relate to research contributions by country including: overall population density; economic factors, e.g., gross domestic product and per capita expenditure and infrastructure that are important drivers supporting research. Tobacco smoking is a global burden with its associated research output spanning both developed and developing countries. The global predictions of smoking-related disease morbidity and mortality is for an increase in smoking-related disease in developing countries, therefore a concerted effort must be made to increase smoking cessation research output within developing countries where smoking rates are highest and burden of disease overrepresented.

This review aimed to identify the number and methodological quality of published smoking cessation research relevant to low-SES and disadvantaged population groups over two time periods (TP1: 2000–2004) and (TP2: 2008–2012) and by country (Australia/New Zealand, United Kingdom, United States/Canada and Other countries).

The hypotheses were that over TP1 and TP2:

(i) Total published research output (reviews, non-data based publications, data-based publications (descriptive, measurement and intervention research)) would have significantly increased;
(ii) The proportion of the total published output that is intervention research would have significantly increased; and
(iii) The methodological quality of the published intervention research would have significantly increased: (a) the proportion of intervention studies using Cochrane Effective Practice Organisation of Care (EPOC)-accepted evaluation designs: randomised controlled trial (RCT), controlled clinical trial (CCT), controlled before and after (CBA) study, or interrupted time series (ITS) would have significantly increased; and (b) the proportion of intervention studies using biochemical verification of self-reported smoking abstinence would have significantly increased.

This review adopted a dichotomous breakdown across two time periods (TP1 and TP2) and allowed for a three year lag time period (between 2005 to 2007) to account for the implementation of the WHO FCTC and to identify whether the WHO FCTC may have impacted smoking cessation research output among low-SES and disadvantaged population groups. This time period took into account that final signatories for the WHO FCTC countries occurred in 2005 and that a number of steps and a substantial timeframe is needed to allow for research output to eventuate, *i.e.*, study conceptualisation, grant proposal and funding approvals, ethics approval, implementation, data collection, data analyses and publication of research.

## 2. Method

### 2.1. Data Sources

#### Search Strategy, Selection of Studies and Coding

**Figure 1:**
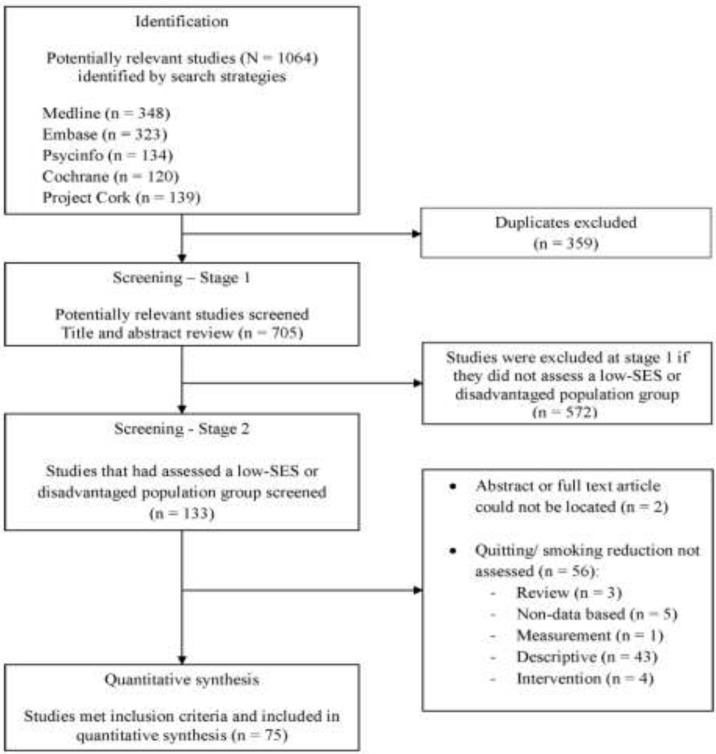
Flowchart of search strategy and selection criteria (2000–2004) using PRISMA guideline.

**Figure 2:**
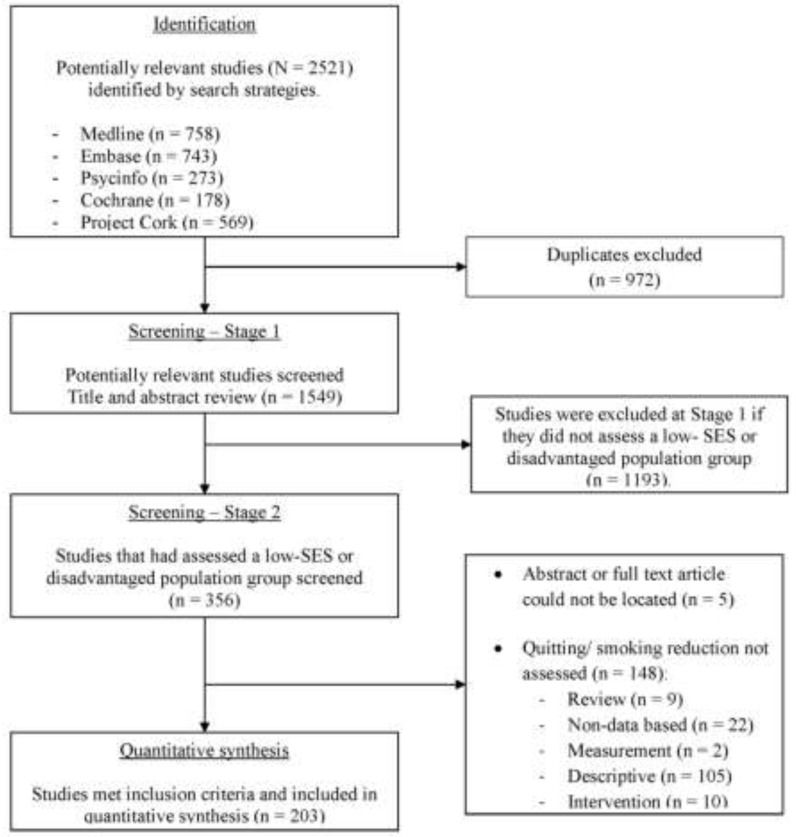
Flowchart of search strategy and selection criteria (2008–2012) using PRISMA guideline.

This review followed the PRISMA statement (See Table A1) for identification, screening, eligibility and inclusion of studies [26]. As summarised in Figure 1 (TP1) and Figure 2 (TP2), Medline, Embase, PsycINFO, The Cochrane Library and Project Cork databases were searched. The search terms are described in full in the Appendix and are accessible online from the *Int. J. Environ Res Public Health* website. *Note:* “smoking cessation” was the search term used in each of the above databases.

### 2.2. Study Selection

The titles and abstracts of citations were examined by author RC for relevance. Studies were included if they were: (1) written in English; (2) published within TP1: 2000–2004 or TP2: 2008–2012; and (3) examined smoking cessation among a low-SES or disadvantaged population group. Smoking cessation studies were included if they provided sustained abstinence, point-prevalence, or binary data relating to quit status (*i.e*., Quit (Yes/No)). Studies that had provided smoking reduction data (*i.e*., number of cigarettes smoked) were also included. Selected publications were coded by: country; year of publication; low-SES or type of disadvantaged population group targeted (homeless, Indigenous, prisoners, at-risk youth, schizophrenia, depression, and other mental illness). Low-SES was defined as persons who: had a low fixed household income; were Medicaid recipients; or lived in impoverished neighbourhoods or disadvantaged communities. The disadvantaged population groups were selected as they are priority population groups with high rates of smoking compared to the general population and, in addition, the burden of illness associated with tobacco use is overrepresented in such populations groups. No limitations were set on type of smoking cessation research included in the review, e.g., behavioural, pharmacological or any policy relevant tobacco control measures, *i.e.*, taxation.

### 2.3. Data Extraction

#### 2.3.1. Publication Volume and Types

Publications were coded into three study types similar to previous reviews investigating public health research output [24,25,27]: (i) Reviews-publications specified as a “review” in the title or abstract or summarising previous research; (ii) Non data-based research-reports of study protocols, commentaries, opinion articles and case studies; and (iii) Data-based research-publications reporting new data or analysis of existing data.

Data-based research publications were further coded by study type (descriptive, measurement or intervention), defined as: (i) Descriptive-data-based epidemiologic research that examined smoking cessation or reduction using qualitative or quantitative methods that had not used an intervention; (ii) Measurement-publications exploring the development of models, psychometric analysis of an instrument or validation of a measurement tool; and (iii) Intervention research-publications that evaluated the effectiveness of smoking cessation intervention(s) aimed at smoking cessation and/or reduction interventions.

#### 2.3.2. Evaluation Designs and Biochemical Verification

Intervention research was classified according to the EPOC data collection checklist for study evaluation design [28]: randomised controlled trial (RCT), controlled clinical trial (CCT), controlled before and after study (CBA) and interrupted time series (ITS). Other evaluation designs were coded as “Non-EPOC”. Intervention studies were also assessed for use of biochemical verification of smoking abstinence. Author RC identified the intervention studies, which were then coded according to their evaluation design and biochemical verification status by RC and PC independently of each other. There was over 95% agreement between coders. Differences were resolved by discussion.

### 2.4. Statistical Analyses

A Poisson regression model was fitted to the generalized estimating equation (GEE) with an autoregressive working correlation matrix specified, in order to account for the effect of clustering in the examined time categories (TP1 and TP2) when counting the number of research publication over the two dichotomous time periods [29]. This type of working correlation matrix was chosen due to the proportional relationship between the numbers of published studies within countries over two separate time categories with the number of total publications. The proportion of intervention studies were compared over the two time categories and stratified by country and by methodological quality as categorised by EPOC adherence or not; in addition to the proportion of intervention studies using biochemical verification of self-reported smoking abstinence. A Wald Chi-square statistic was used to assess the change in the proportion of intervention studies across countries and for methodological rigour between the two time categories. SPSS 22 was used to address each of these outcomes, and to model the Poisson regression within the GEE.

## 3. Results

### 3.1. Data Synthesis

#### Identification of Studies

The literature search results are summarised in Figure 1 (TP1) and Figure 2 (TP2). In TP1, 1064 articles were identified, of which 75 articles met the inclusion criteria. In TP2, 2521 articles were identified, of which 203 met the inclusion criteria (See Figure 1 and Figure 2).

### 3.2. Volume of Research

The Poisson regression was fitted to the GEE, with time and country as predictors of research output. The results showed that there were no significant main or interaction effects of time and country on overall research output when the effects of clustering are accounted for in the analysis. However it must be noted that research output did show a trend of overall increase in the number of publications across both time periods in all countries from 75 studies in TP1 (27% of all studies) to 203 studies in TP2 (73% of all studies). Of those, as indicated in Table 1, 43 (57% of total in TP1) studies in TP1 and 112 (55% of TP2) in TP2 were data based.

**Table 1:** Research output by country, publication type and time period represented by number of publications and proportion (%) output.

Country	TP1		TP2	
	Descriptive ^a^	Intervention	Descriptive	Intervention
Australia/New Zealand	1 (25%)	3 (75%)	4 (31%)	9 (69%)
United States/Canada	6 (19%)	26 (81%)	31 (36%)	54 (64%)
United Kingdom	2 (40%)	3 (60%)	0 (0%)	1 (100%)
Other	1 (50%)	1 (50%)	2 (15%)	11 (85%)
Total	10	33	37	75

**^a^** No measurement publications were identified.

Data-based (descriptive or intervention) research output also increased between TP1 (*n* = 43 studies) and TP2 (*n* = 112). However, the proportion of data-based research that focussed on interventions did not increase between TP1 (77%−33/43) and TP2 (67%−75/112). This pattern of intervention research output was consistent for Australia (*χ*² = 0.05, *p* = 0.83), the United States and Canada (*χ*² = 3.4, *p* = 0.07), the United Kingdom (*χ*² = 0.6, *p* = 0.44) and other countries (*χ*² = 1.3, *p* = 0.26).

### 3.3. Specific Low-SES and Disadvantaged Populations by Country

Table 2 shows no statistically significant change in the population groups studied from TP1 to TP2 (*χ*² = 4.8, *p* = 0.3). Note: due to small cell sizes, the mental illness category comprises schizophrenia, depression and other mental health disorders, and homeless was combined with low-SES. This pattern was consistent across countries.

**Table 2:** Research output by country and population group across time periods represented by number of publications and proportion (%) output.

Country	TP1						TP2					
	Low-SES/ Homeless	Mental Illness	Prisoners	At-risk Youth	Indigenous	Total	Low-SES/ Homeless	Mental Illness	Prisoners	At-risk Youth	Indigenous	Total
Australia/ New Zealand	0 (0%)	5 (45%)	2 (18%)	0 (0%)	4 (36%)	11	5 (15%)	12 (35%)	4 (12%)	0 (0%)	13 (38%)	34
United States/ Canada	11 (21%)	29 (56%)	1 (2%)	7 (13%)	4 (8%)	52	33 (24%)	76 (55%)	7 (5%)	7 (5%)	16 (12%)	139
United Kingdom	8 (89%)	0 (0%)	1 (11%)	0 (0%)	0 (0%)	9	2 (29%)	4 (57%)	1 (14%)	0 (0%)	0 (0%)	7
Other	0 (0%)	3 (100%)	0 (0%)	0 (0%)	0 (0%)	3	5 (22%)	18 (78%)	0 (0%)	0 (0%)	0 (0%)	23
Total	19 (25%)	37 (49%)	4 (5%)	7 (9%)	8 (11%)	75	45 (22%)	110 (54%)	12 (6%)	7 (3%)	29 (14%)	203

### 3.4. Proportion of Intervention Studies

Table 3 shows that the number of both EPOC and non-EPOC evaluation designs increased over time: 21 (TP1) to 44 (TP2); and 12 (TP1) to 31 (TP2), respectively. Within the EPOC studies, the proportion that were RCTs remained comparable from T1 (64%) to T2 (57%) (*χ*² = 0.24, *p* = 0.63).

**Table 3:** Number of intervention studies and respective EPOC evaluation design used by country represented by number of publications and proportion (%) output.

Country	TP1				TP2			
	EPOC		Non-EPOC	Total	EPOC		Non-EPOC	Total
	RCT	CCT			RCT	CCT		
Australia/New Zealand	1 (33%)	0 (0%)	2 (67%)	3	2 (22%)	0 (0%)	7 (78%)	9
United States/Canada	17 (65%)	0 (0%)	9 (35%)	26	34 (63%)	1 (2%)	19 (35%)	54
United Kingdom	2 (67%)	0 (0%)	1 (33%)	3	0 (0%)	0 (0%)	1 (100%)	1
Other	1 (100%)	0 (0%)	0 (0%)	1	7 (64%)	0 (0%)	4 (36%)	11
Total	21 (64%)	0 (0%)	12 (36%)	33	43 (57%)	1 (1%)	31 (41%)	75

There were no EPOC endorsed designs other than RCTs and CCTs.

### 3.5. Methodological Quality of Intervention Studies

#### 3.5.1. Evaluation Designs

Although the number of intervention studies using EPOC-accepted evaluation design across population groups had increased from 21 in TP1 to 44 in TP2, the proportion of intervention studies examining each population group (see Table 4) remained consistent from TP1 to TP2 (*χ*² = 3.97, *p* = 0.41).

**Table 4:** Number of intervention studies using EPOC accepted evaluation design by country and population group represented by number of publications and proportion (%) output.

Country	TP1						TP2					
	Low-SES/ Homeless	Mental Illness	Prisoners	At-risk Youth	Indigenous	Total	Low-SES/ Homeless	Mental Illness	Prisoners	At-risk Youth	Indigenous	Total
Australia/ New Zealand	0 (0%)	1 (100%)	0 (0%)	0 (0%)	0 (0%)	1	0 (0%)	1 (50%)	0 (0%)	0 (0%)	1 (50%)	2
United States/ Canada	4 (24%)	9 (53%)	0 (0%)	3 (18%)	1 (6%)	17	9 (26%)	19 (54%)	4 (11%)	2 (6%)	1 (3%)	35
United Kingdom	2 (100%)	0 (0%)	0 (0%)	0 (0%)	0 (0%)	2	0 (0%)	0 (0%)	0 (0%)	0 (0%)	0 (0%)	0
Other	0 (0%)	1 (100%)	0 (0%)	0 (0%)	0 (0%)	1	1 (14%)	6 (86%)	0 (0%)	0 (0%)	0 (0%)	7
Total	6 (29%)	11 (52%)	0 (0%)	3 (14%)	1 (5%)	21	10 (23%)	26 (59%)	4 (9%)	2 (5%)	2 (5%)	44

#### 3.5.2. Use of Biochemical Verification

Of the intervention studies (33 in TP1 and 75 in TP2) the proportion that used biochemical verification of smoking status declined significantly from TP1 88% to 64% at TP2 (*χ*² = 6.39, *p* = 0.01). There was a significant difference in the use of biochemical verification by EPOC design type: of the 65 RCTs or CCTs, 82% (*n* = 53 studies) used some form of biochemical verification, compared to 56% (*n* =24 studies) of the 43 “Non-EPOC” evaluation designs (*χ*² = 8.37, *p* < 0.01).

## 4. Discussion

### 4.1. Summary of Findings

While the total number of published studies increased from TP1 to TP2 (confirmation of hypothesis 1), the proportion of intervention research did not significantly change over time (contrary to hypothesis 2), nor did the proportion of intervention research that had used an EPOC-accepted evaluation design or any increase in biochemical verification of self-reported smoking status (contrary to hypothesis 3).

### 4.2. Increasing Output of Methodologically Rigorous Intervention Research

#### Using Accepted EPOC Evaluation Designs

Although it is acknowledged that RCTs are not always feasible, EPOC has identified a number of alternatives that provide sufficiently rigorous evidence [30,31]. Such alternative research designs (e.g., multiple baseline design) have an added benefit of being more cost- and time-efficient than RCTs and their integration into policy may be more feasible [30,32]. The apparent lack of proportional increase in implementing these alternatives, or RCTs, in smoking cessation interventions for low-SES and disadvantaged population groups should be addressed as a priority in future smoking cessation studies.

Several potential options are available to increase the output of methodologically robust intervention studies. First, incentivising and providing strategic funding towards EPOC-accepted evaluation designs may assist in increasing such outputs [24,25]. There may be significant value in research granting bodies prioritising the allocation of funding towards intervention studies using methodologically sound designs. At an institutional level, performance indicators of researchers could be shifted towards a focus on methodological quality of researchers’ outputs and their potential to impact on health care policy [24,25]. If research institutions incentivised and rewarded publications accepted in Cochrane Database of Systematic Reviews, there may be an increased impetus for academics to develop methodologically rigorous interventions [24,25]. In addition, journal publishers could reorient their publication criteria to have a greater focus on methodological adequacy of studies rather than significance of results [24,25]. There have been calls for the establishment of research centres and collaborations designed to meet the distinct needs of distinct population groups in a co-ordinated manner [33,34,35]. Such a strategic and focused research effort may present a number of positive flow on effects including: pooling of funding and resources; knowledge gathering and translation; access to multidisciplinary advice; promotion of a high-quality research agenda; and expanding and developing partnerships with community organisations and stakeholders to increase recruitment and retention [33].

It is also important consumer advisory groups (CAGs) are involved in priority-driven research agendas and the direction of future intervention research among low-SES and disadvantaged population groups. CAGs can provide valuable information on effective and time efficient strategies for recruitment, retention, and intervention delivery [36]. At a provider-level, it is also recognised that staff at health and community organisations may not have the confidence or empowerment to implement intervention research [37]. A concerted effort needs to be made to encourage CAGs, health care organisations, staff and health practitioners to partner, engage and help to foster and increase intervention research output.

### 4.3. Increasing the Use of Biochemical Verification of Self-Reported Abstinence

In addition to increasing the use of rigorous evaluation designs, this review highlights that improvements in the reporting of biochemical verification of self-reported smoking may be warranted. The use of biochemical verification is central to determining the accuracy of smoking abstinence claims and is used in nearly all publications in high-impact journals [38]. It is largely acknowledged that among special populations or for clinic-based interventions biochemical verification does add higher precision [39,40]. Self-reported smoking prevalence using standard methods has been shown to underestimate national smoking prevalence estimates across countries with cotinine concentrations in misclassified non-smokers typically indicative of high levels of smoking and/or exposure to smoke [41]. There is a need for correction of self-reported estimates using biochemical markers, as success or failure of tobacco control measures and smoking cessation intervention research is typically measured by changes in smoking rates of a few percentage points and consideration of even low error rates are extremely important [41]. The fact that only 70% of intervention studies identified in this review used biochemical verification, and its significant decline in use from TP1 to TP2, suggests it could be used more frequently. Allowing for biochemical verification to verify health behaviours such as smoking cessation is of particular importance in countering the Hawthorne effect that may lead to misclassification due to the reluctance of participants to report that they have failed to cease smoking [38,42]. A double blind controlled study may still not counter all three components of this phenomenon since controlling for “treatment”, “observation” and “experimenter” bias may not be feasible, practical or even desired since these effects may in fact improve the saliency of the intervention [43]. Biochemical verification would provide insights into this often neglected but important phenomenon. However, careful consideration must be made about the feasibility of biochemical verification outside of clinically-oriented behavioral or pharmacological interventions as for some interventions, e.g., taxation, media and other policy interventions biochemical verification may not be feasible.

### 4.4. Study Limitations

Despite using an extensive search strategy across several databases, including citations from developing and developed countries, it is possible that some eligible studies were excluded. Hand searching, in addition to electronic searching, is recommended as an additional search strategy [44] but the large number of citations identified across both time periods (*n* = 278 citations) meant that this task was neither practical nor feasible. A combination of explode and focus commands on subject headings were used to achieve a high level of sensitivity and specificity for the literature search. The * symbol was used before the search statement “smoking cessation” to ensure we identified results where this term was a major topic or focus. Further, it is acknowledged that the use of additional terms, e.g., “tobacco use cessation” and not limiting the search to “humans” and “English language” may have provided a more sensitive search string. This may have meant that some studies where smoking cessation was not a primary focus were missed but the likelihood of any such bias would have been equal across country and low-SES and disadvantaged population groups studied. This review used number of published articles as a proxy for research output, however, citation analysis is an alternative measure that could be used to assess research quality in the future. EPOC evaluation design categories were used as the measure of methodological quality of studies, rather than applying additional criteria. Nonetheless, the relatively high proportion (40%) that did not meet basic EPOC evaluation design criteria is concerning because it is difficult to be confident of a causal relationship between the intervention and the outcome, irrespective of the quality of other design features. This problem has also been identified in other reviews examining research output in indigenous health, low-SES groups, quality of life and other public health research [24,25,27,45,46].

## 5. Conclusions

The purpose of this review was to identify the pattern of research output among low-SES and disadvantaged population groups and to encourage debate on how research efforts should be directed to improve knowledge and increase output of methodologically sound smoking cessation research. The current review findings coupled with a paucity of methodological rigorous intervention research aimed at low-SES and disadvantaged populations groups indicates that tobacco-related health inequalities, high rates of smoking, and high economic costs may continue in the foreseeable future. The development and implementation of high quality intervention research among low-SES and disadvantaged population groups remains a high clinical and economic priority. Both funding bodies and researchers alike must critically reflect on the suboptimal output of intervention research. Identifying mechanisms to effect change may be difficult, but it is of utmost importance to allocate research resources that contribute most effectively to health gain through the application of evidence-based practice [25].

## Appendix

### Search Terms for Identification and Selection of Studies

For MEDLINE we separately searched from 2000 to 2004 and 2008 to 2012 limited to “English language” and “Humans” using the following Medical Subject Heading (MeSH) terms and keywords: (exp ***** Smoking Cessation/) and (exp Socioeconomic Factors/or exp Poverty/or exp Vulnerable Populations/or exp Homeless Persons/or exp Mentally Ill Persons/or exp Mental Health/or exp Schizophrenia/or exp Anxiety/or exp Depression/or exp Prisons/or exp Prisoners/or exp Adolescent Behavior/or exp Juvenile Delinquency/or exp Oceanic Ancestry Group/or exp Indians, Central American/or exp Indians, North American/or exp Inuits/or “first nation$”.mp. or “aborigin$”.mp or “maori”.mp).

For EMBASE we separately searched from 2000 to 2004 and 2008 to 2012 limited to “English language” and “Humans” using the following Emtree terms and keywords: (exp ***** smoking cessation/) and (exp socioeconomics/or exp social class/or exp social status/or exp poverty/or exp lowest income group/or exp vulnerable population/or exp homelessness/or exp mental patient/or exp mental health/or exp schizophrenia/or exp anxiety/or exp depression/or exp prison/or exp prisoner/or exp adolescence/or exp juvenile/or exp Aborigine/or exp American Indian/or exp indigenous people/or exp Eskimo/or exp Maori/or first nation$.mp. or aborigin$.mp).

For PsycINFO we separately searched from 2000 to 2004 and 2008 to 2012 limited to “English language” and “Humans” using the following PsycINFO terms and keywords: (exp ***** smoking cessation/) and (exp Socioeconomic Status/or exp Family Socioeconomic Level/or exp Social Class/or exp Lower Income Level/or exp Poverty/or exp Poverty Areas/or exp At Risk Populations/or exp Homeless/or exp Homeless Mentally Ill/or exp Mental Health/or exp Psychiatric Patients/or exp Schizophrenia/or exp Anxiety/or exp Major Depression/or exp Prisons/or exp Prisoners/or exp Juvenile Delinquency/or exp Indigenous Populations/or exp American Indians/or exp Inuit/or exp Alaska natives/or first nation$.mp. or aborigin$.mp. or maori.mp).

For The Cochrane Library we separately searched from 2000 to 2004 and 2008 to 2012 limited to “English language” and “Humans” using the following Medical Subject Heading (MeSH) terms and keywords: (smoking cessation) and (Socioeconomic factors or Vulnerable populations or Homeless persons or Mentally ill persons or Mental health or Schizophrenia or Anxiety or Depression or Prisons or Prisoners or Adolescent behaviour or Juvenile delinquency or Oceanic ancestry group or American Native Continental Ancestry Group or Aborigin ***** or Indigenous or Maori).

For Project Cork we separately searched from 2000 to 2004 and 2008 to 2012 limited to “English language” and “Humans” using the following Project Cork terms and keywords: (smoking cessation) and (sociological aspect or homeless or chronic mental illness or anxiety or anxiety disorder or mood disorder or depression or schizophrenia or prisoners or criminal justice system or adolescents or native peoples or Native Americans or Alaska Natives).

**Table A1:** PRISMA 2009 checklist.

Section/Topic	#	Checklist Item	Reported on Page #
**TITLE**			
Title	1	Identify the report as a systematic review, meta-analysis, or both.	1
**ABSTRACT**			
Structured summary	2	Provide a structured summary including, as applicable: background; objectives; data sources; study eligibility criteria, participants, and interventions; study appraisal and synthesis methods; results; limitations; conclusions and implications of key findings; systematic review registration number.	1-2
**INTRODUCTION**			
Rationale	3	Describe the rationale for the review in the context of what is already known.	2-4–5
Objectives	4	Provide an explicit statement of questions being addressed with reference to participants, interventions, comparisons, outcomes, and study design (PICOS).	3-4
**METHODS**			
Protocol and registration	5	Indicate if a review protocol exists, if and where it can be accessed (e.g., Web address), and, if available, provide registration information including registration number.	N/A
Eligibility criteria	6	Specify study characteristics (e.g., PICOS, length of follow-up) and report characteristics (e.g., years considered, language, publication status) used as criteria for eligibility, giving rationale.	6
Information sources	7	Describe all information sources (e.g., databases with dates of coverage, contact with study authors to identify additional studies) in the search and date last searched.	6
Search	8	Present full electronic search strategy for at least one database, including any limits used, such that it could be repeated.	Online appendix
Study selection	9	State the process for selecting studies (*i.e*., screening, eligibility, included in systematic review, and, if applicable, included in the meta-analysis).	6 + Figures
Data collection process	10	Describe method of data extraction from reports (e.g., piloted forms, independently, in duplicate) and any processes for obtaining and confirming data from investigators.	N/A
Data items	11	List and define all variables for which data were sought (e.g., PICOS, funding sources) and any assumptions and simplifications made.	6–7
Risk of bias in individual studies	12	Describe methods used for assessing risk of bias of individual studies (including specification of whether this was done at the study or outcome level), and how this information is to be used in any data synthesis.	7. EPOC checklist
Summary measures	13	State the principal summary measures (e.g., risk ratio, difference in means).	7
Synthesis of results	14	Describe the methods of handling data and combining results of studies, if done, including measures of consistency (e.g., I^2^) for each meta-analysis.	7
Risk of bias across studies	15	Specify any assessment of risk of bias that may affect the cumulative evidence (e.g., publication bias, selective reporting within studies).	12
Additional analyses	16	Describe methods of additional analyses (e.g., sensitivity or subgroup analyses, meta-regression), if done, indicating which were pre-specified.	N/A
**RESULTS**			
Study selection	17	Give numbers of studies screened, assessed for eligibility, and included in the review, with reasons for exclusions at each stage, ideally with a flow diagram.	8 + Figures
Study characteristics	18	For each study, present characteristics for which data were extracted (e.g., study size, PICOS, follow-up period) and provide the citations.	7
Risk of bias within studies	19	Present data on risk of bias of each study and, if available, any outcome level assessment (see item 12).	7. EPOC checklist
Results of individual studies	20	For all outcomes considered (benefits or harms), present, for each study: (a) simple summary data for each intervention group (b) effect estimates and confidence intervals, ideally with a forest plot.	N/A
Synthesis of results	21	Present results of each meta-analysis done, including confidence intervals and measures of consistency.	N/A
Risk of bias across studies	22	Present results of any assessment of risk of bias across studies (see Item 15).	N/A
Additional analysis	23	Give results of additional analyses, if done (e.g., sensitivity or subgroup analyses, meta-regression (see Item 16)).	N/A
**DISCUSSION**			
Summary of evidence	24	Summarize the main findings including the strength of evidence for each main outcome; consider their relevance to key groups (e.g., healthcare providers, users, and policy makers).	10–13
Limitations	25	Discuss limitations at study and outcome level (e.g., risk of bias), and at review-level (e.g., incomplete retrieval of identified research, reporting bias).	12
Conclusions	26	Provide a general interpretation of the results in the context of other evidence, and implications for future research.	13
**FUNDING**			
Funding	27	Describe sources of funding for the systematic review and other support (e.g., supply of data); role of funders for the systematic review.	13

*From:* Moher, D.; Liberati, A.; Tetzlaff, J.; Altman, D.G.; The PRISMA Group. Preferred Reporting Items for Systematic Reviews and Meta-Analyses: The PRISMA Statement. *PLoS Med.* **2009**, *6*, e1000097, doi:10.1371/journal.pmed1000097; For more information, visit: www.prisma-statement.org.
